# Position paper: management of perforated sigmoid diverticulitis

**DOI:** 10.1186/1749-7922-8-55

**Published:** 2013-12-26

**Authors:** Frederick A Moore, Fausto Catena, Ernest E Moore, Ari Leppaniemi, Andrew B Peitzmann

**Affiliations:** 1 Acute Care Surgery, University of Florida, 1600 Southwest Archer Road, PO Box 100108, Gainesville, FL 32610-0108, USA; 2 Emergency Surgery Department, Parma University Hospital, Via Cracvia 23, Bologna 40139, Italy; 3 University of Colorado Health Science Center, Denver Health Science Center, 777 Bannock Street, Denver, CO 80204-4507, USA; 4 Department of Abdominal Surgery, University of Helsinki, Haartmaninkatu 4, PO Box 340, Meilahi Hospital, FIN-00029 HUS, Helsinki, HUS 00290, Finland; 5 University of Pittsburgh, F-1281, UPMC-Presbyterian, Pittsburgh, PA 15213, USA

**Keywords:** Complicated diverticulitis, Hartmann’s procedure, Primary resection anastomosis, Laparoscopic lavage and drainage, Percutaneous drainage

## Abstract

Over the last three decades, emergency surgery for perforated sigmoid diverticulitis has evolved dramatically but remains controversial. Diverticulitis is categorized as uncomplicated (amenable to outpatient treatment) versus complicated (requiring hospitalization). Patients with complicated diverticulitis undergo computerized tomography (CT) scanning and the CT findings are used categorize the severity of disease. Treatment of stage I (phlegmon with or without small abscess) and stage II (phlegmon with large abscess) diverticulitis (which includes bowel rest, intravenous antibiotics and percutaneous drainage (*PCD*) of the larger abscesses) has not changed much over last two decades. On the other hand, treatment of stage III (purulent peritonitis) and stage IV (feculent peritonitis) diverticulitis has evolved dramatically and remains morbid. In the 1980s a two stage procedure (1^st^ - segmental sigmoid resection with end colostomy and 2^nd^ - colostomy closure after three to six months) was standard of care for most general surgeons. However, it was recognized that half of these patients never had their colostomy reversed and that colostomy closure was a morbid procedure. As a result starting in the 1990s colorectal surgical specialists increasing performed a one stage primary resection anastomosis (*PRA*) and demonstrated similar outcomes to the two stage procedure. In the mid 2000s, the colorectal surgeons promoted this as standard of care. But unfortunately despite advances in perioperative care and their excellent surgical skills, *PRA* for stage III/IV diverticulitis continued to have a high mortality (10-15%). The survivors require prolonged hospital stays and often do not fully recover. Recent case series indicate that a substantial portion of the patients who previously were subjected to emergency sigmoid colectomy can be successfully treated with less invasive nonoperative management with salvage *PCD* and/or laparoscopic lavage and drainage. These patients experience a surprisingly lower mortality and more rapid recovery. They are also spared the need for a colostomy and do not appear to benefit from a delayed elective sigmoid colectomy. While we await the final results ongoing prospective randomized clinical trials testing these less invasive alternatives, we have proposed (based primarily on case series and our expert opinions) what we believe safe and rationale management strategy.

## Introduction

This position paper updates the literature related to the management of perforated sigmoid diverticulitis with the goals of identifying a) key management decisions, b) alternative management options and c) gaps in our knowledge base that can be targeted in a future emergency surgery research agenda [1,2]. From this we have created a decision making algorithm that can be modified based on evolving evidence and local resources to guide institutional practices. This manuscript will provide the basis for a future evidence based guideline (EBG) that will be developed and endorsed by the World Society of Emergency Surgery and published in the World Journal of Emergency Surgery. We envision that the EBG recommendations will be graded based on the level of evidence and will identify the resources needed to provide optimal care. Recognizing the tremendous variability in hospital resources available worldwide, this optimal resource information will be used to designate levels of acute care surgery hospitals (similar to trauma centers). This designation process will be used to leverage hospitals to upgrade their resources to optimize their emergency surgery capabilities.

## Background and significance

### Pathogenesis

Diverticular disease is common affecting over 50% of men and women older than 65 years. Diverticulitis is inflammation of the colon that occurs as a result of perforation of a diverticulum almost exclusively in the sigmoid colon and incidence is estimated to be 3.4 to 4.5 per 100,000 people per year [3-6]. Diverticulitis is known as the *disease of the industrial revolution*, since there are no reports or pathologic specimens documenting evidence of diverticular disease prior to the 1900s [7]. In the late 1800s, the process of roller-milling wheat was introduced which removes two thirds of the fiber content of wheat. Coincident with this implementation, diverticulosis was observed in the first decade of the 1900s. It is now known that a diet low in fiber is a contributing factor in the development of diverticular disease [7-9]. In a study of nearly 48,000 US men, a low-fiber diet increased the risk of symptomatic diverticular disease by two- to threefold over a 4-year period [10]. In addition to low dietary fiber, alterations in colonic intraluminal pressures have been shown in patients with diverticular disease. Although resting intraluminal pressures between diverticular disease patients and controls do not differ significantly, higher pressures have been demonstrated in segments of colon with diverticula [11]. In addition, later studies indicate increased colonic motility, as assessed by the number and amplitude of bowel wall contractions, in the sigmoid colon of patients with diverticular disease [12-14]. Therefore, both a low-fiber diet and colonic dysmotility have been implicated in the pathogenesis of diverticular disease.

### Treatment options

These are based upon the stage of disease. Table 1 depicts a scoring system that subdivides diverticulitis based upon the extent of disease identified on computerized tomography (CT) scanning. The traditional Hinchey classification was developed before routine CT scanning [15] and we have modified it slightly to reflect contemporary management decisions that are based on CT scan findings. Most clinicians are comfortable treating patients stage IA and IB diverticulitis with intravenous (IV) antibiotics and bowel rest. They will also readily opt for interventional radiology percutaneous drainage (*PCD*) in patients with stage IIB disease as long as the patients do not have severe sepsis/septic shock (SS/SS). However, there is considerable controversy over what is the best option for patients who present with stage III and IV diverticulitis who have signs of SS/SS. The treatment options for these patients are described below:

**Table 1:** Perforated sigmoid diverticulitis score

**Stage**	**CT scan findings**
IA	Phelogmon with no abscess
IB	Phlegmon with abscess ≤ 4 cm
II	Phlegmon with abscess > 4 cm
III	Purulent pertonitis (no hole in colon)
IV	Feculent pertonitis (persistent hole in colon)

### Three stage procedure

While diverticulosis was initially regarded as a pathologic curiosity, the first colon resection for perforated diverticulitis was reported by Mayo in 1907 [16]. However, a subsequent report from the Mayo clinic in 1924, concluded that acute resection accentuated the infection resulting in a prohibited high mortality [17]. They recommended a colostomy with distal irrigation and then delayed resection when the patient condition improved. Over the next 20 years, a variety of procedures were performed for perforated diverticulitis. In 1942 the Massachusetts General Hospital reported their experience with these different procedures and concluded that the best outcomes were achieved with proximal diverting colostomy and then resection of the diseased colon in three to six months after the inflammation had resolved [18]. Thereafter the three stage procedure became the standard of care: 1^st^ - diverting transverse colostomy and drainage; 2^nd^ - definitive resection and colostomy after three to six months and 3^rd^ - colostomy closure after three to six months.

### Two stage procedure

After the introduction of perioperative antibiotics and improved perioperative care, case series emerged starting in the late 1950s that demonstrated that in select circumstances the diseased colon could be safely resected at the 1^st^ operation. The two stage procedure: 1^st^ - segmental sigmoid resection with end colostomy [i.e. the Hartmann’s procedure (*HP*) originally described Henri Hartmann in 1921 for treatment of colorectal cancer] [19] and 2^nd^ - colostomy closure after three to six months was increasingly practiced and became standard of care by the 1980s. This approach was supported by a study published in 1984 which combined patient data from 36 case series published since the late 1950s [20]. The study include a total of 821 cases of diverticulitis with purulent (i.e. stage III disease) or feculent (i.e. stage IV disease) peritonitis of which 316 patients underwent a *HP* (with a mortality of 12%) compared to the 505 patients who underwent diverting colostomy with no resection (with a mortality of 29%). While these retrospective case series suffer from selection bias in that the less healthy patients were more likely to undergo a diverting colostomy with no resection, this report established that a substantial portion of patients can undergo an emergency *HP* with an acceptable mortality. Additionally, acute resection avoided missing a colon cancer (which occurs in up to 3% of cases) and decreased morbidity because up to 20% of the non-resected patients developed a fistula. Interestingly, there were two subsequent prospective randomized trials (PRTs) that showed divergent results. In a single center Swedish PRT, of 46 patients with stage III purulent peritonitis, 25 patients who underwent a *HP* (with 24% mortality) compared to 21 patients who underwent colostomy with no resection (with 0% mortality) [21]. In a multicenter French PRT of 103 patients with purulent or feculent peritonitis, 55 patients underwent a *HP* and had a < 2% rate of post-operative sepsis with a mortality of 23% [22]. In contrast, 48 patients underwent diverting colostomy with no resection (with suture closure of the hole in the stage IV cases) had a 20% rate of post-operative sepsis with a similar mortality of 18%. As a result of these and other data, the colorectal surgical specialists published an EBG in 2000 in which they concluded that the procedure of choice for perforated diverticulitis was a *HP*[23]. However, with the recognition up to half of the patients who underwent a *HP* never had their colostomy reversed and that colostomy closure was a morbid procedure, many colorectal surgeons performed a primary anastomosis in select cases.

### Primary resection with anastomosis (PRA)

A 2006 meta-analysis [that included 15 case series (13 retrospective)] indicated that mortality was significantly lower and there was a trend towards fewer surgical complications in patients who underwent *PRA* with or without a proximal diverting loop ileostomy compared those who underwent a *HP* for perforated diverticulitis [24]. Again, while this review suffers from a selection bias where the less healthy patients were more likely to undergo a HP, it does document that emergency *PRA* in select patients has a low anastomotic leak rate (~6%) and that in the sicker patients (stage > II subset) *PRA* and *HP* had equivalent mortality (14.0 vs. 14.4%). Additionally, it was recognized that 85% of patients with *PRA* and proximal loop ileostomy had subsequent stomal closure [25]. As a result of these data, the colorectal surgical specialists updated their EBG in 2006 and recommended emergent definitive sigmoid resection for perforated diverticulitis with peritonitis but concluded that an acceptable alternative to the *HP* (i.e. colostomy) is primary anastomosis [26]. The precise role of proximal ileostomy diversion after *PRA* remains unsettled.

### Laparoscopic lavage and drainage (LLD)

**Figure 1:**
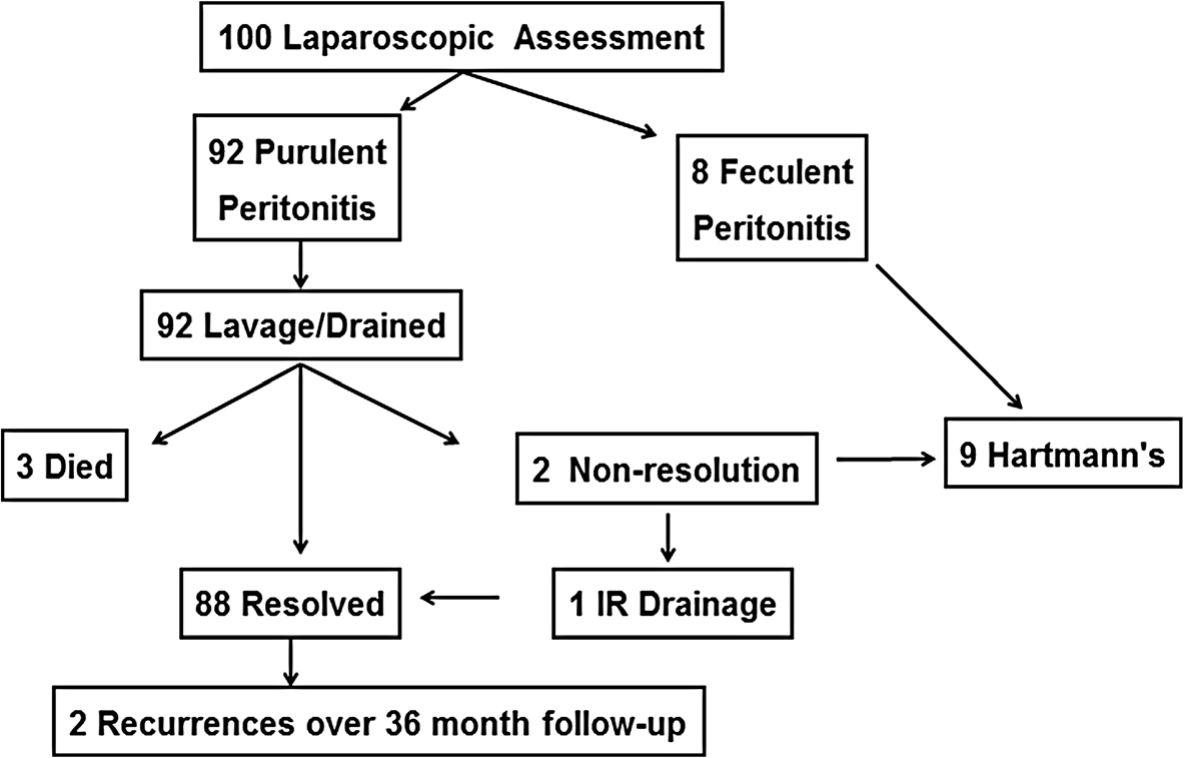
Experience with laporoscopic lavage and drainage.

Interestingly, as the colorectal surgical specialists progressively endorsed a more aggressive approach, starting in 1996, there have been 18 case series involving 806 patients that document surprisingly better outcomes with simple *LLD*[27,28]. In 2008 Myers et al. reported the largest series to date with compelling results (Figure 1) [29]. Out of 1257 patients admitted for diverticulitis over seven years, 100 (7%) had peritonitis with evidence of free air on x-ray or CT scan. These patients were resuscitated, given a third generation cephalosporin and flagyl and then taken emergently to the OR for laparoscopy. Eight were found to have stage IV disease and underwent a *HP*. The remaining 92 patients underwent *LLD*. Three (3%) of these patients died (which much lower than reported for *PRA* or *HP*). An additional two patients had non-resolution, one required an *HP*, and the other had further *PCD*. Overall, 88 of the 92 *LLD* patients had resolution of their symptoms. They were discharged to home and did not undergo an elective resection. Over the ensuing 36 months, there were only two recurrences. Another recent study by Liang et al. associates supports *LLD*[30]. They reviewed 88 cases of diverticulitis (predominantly stage III) treated laparoscopically of which 47 were treated by *LLD* and 41 by laparoscopic *HP* (see Table 2) [30]. Again *LLD* appeared effective for source control and had better outcome than a laparoscopic *HP*. Interesting, they treated 5 cases of stage IV disease with *LLD* combined with laparoscopic closure of the sigmoid colon perforation. Most recently the Dutch have reviewed their experience with *LLD* in 38 patients and reported notably less impressive outcomes [28]. In 31 patients the *LLD* controlled the sepsis. These patients had low mortality (1 died), acceptable morbidity and relatively rapid recovers. However, in the remaining 7 patients *LLD* did not control abdominal sepsis, two died of multiple organ failure (MOF) and 5 required further surgical interventions (3 *HP*s, 1 diverting stoma and 1 perforation closure). One of these died from aspiration and the remaining four experienced prolonged complicated recoveries. These authors concluded that patient selection is of utmost importance. They believe it is contraindicated in stage IV disease. Additionally they noted that patients with stage III disease who have multiple co-morbidities, immunosuppression, a high C reactive protein level and/or a high Mannheim Peritonitis Index are at high risk of failure and concluded that a *HP* as a first step is the best option in these patients.

**Table 2:** Laparoscopic lavage and drainage (LLD) compared to laparoscopic hatman’s procedure (LHP)

	**LLD**	**LHP**	**p value**
# of patient	47	41	
OR time (minutes)	100 ± 40	182 ± 55	0.001
Conversion	2%	15%	0.05
Complications	4%	13%	0.05
Mortality	0%	2.4%	ns
Hospital stay (days)	6.6 ± 2.4	16.6 ± 10	0.01
Colostomy closure	na	72%	na
Elective resection	45%	na	na

### Nonoperative management (NOM)

More recently, Costi et al. added more controversy to management options when they reported their experience with *NOM* of 39 hemodynamically stable patients with stage III diverticulitis [31]. Three (8%) required an emergency operation because of clinical deterioration and underwent an *HP*. Seven (18%) required later CT-guided *PCD* of abscesses, while amazingly 29 (74%) required no early operative intervention and hospital mortality was zero. Half of the discharged patients underwent a delayed elective sigmoid resection and of the remaining half, five had recurrent diverticulitis successfully treated medically (with later elective resection). Of note, patients who underwent delayed elective resection experienced higher than expected morbidity leading the authors to conclude that perhaps delayed resection is not necessary and causes more harm than good. It is surmised with resolution of an acute perforation; local fibrosis prevents the recurrent perforation of the diverticulum. Dr Costi has cautioned that it is imperative to differentiate stage III from stage IV disease. They accomplish this by using a CT scan protocol that utilizes rectal contrast and if the any extravasation is seen, the patient is not a candidate for *NOM*.

### Staged laparotomy

The concept of a *planned relaparotomy* for fulminant peritonitis has been debated for over thirty years. Reoperations are performed every 48 hours for “washouts” until the abdomen is free of ongoing peritonitis and then the abdomen is closed. This supposedly prevents and/or provides early treatment for secondary infections thus decreasing late MOF and deaths. The downside of the *planned relaparotomy* approach is increased resource utilization and the increased potential risk for gastrointestinal fistulas and delayed hernias. The alternative is referred to as *relaparotomy on-demand* where relaparotomy is performed for clinical deterioration or lack of improvement. The potential downside to this approach is harmful delays in diagnosing secondary abdominal infections and the presence of more dense adhesions if there is a need to re-operate. Over the years there have been eight case series that have offered conflicting results regarding the impact of these strategies on outcome. A meta-analysis of these data concluded *relaparotomy on-demand* was the preferred approach in patients with APACHE II <10 [32]. Furthermore, a recent PRT by van Ruler et. al. in patients with APACHE II >10 indicates that the practice of *planned relaparotomy* offered no clinical advantage over *relaparotomy on-demand* and was associated with substantial increases in expenditure of hospital resources [33].

### Damage control laparotomy (DCL)

In the early 1980’s trauma surgeons recognized when they operated in the setting of the “bloody viscous cycle” of acidosis, hypothermia and coagulopathy, operating room (OR) mortality from bleeding was unacceptably high [34]. This prompted the develop of the concept of an abbreviated laparotomy using gauze packing to stop bleeding combined temporary abdominal closure (*TAC*) and triage to the ICU with the intent of optimizing physiology [35]. The patient is taken back to the OR after 24–48 hours for definitive treatment of injuries and abdominal closure. This concept was initially promoted for major liver injuries as a way to avoid major liver resections but was soon extended to all emergency trauma laparotomies [36]. Over the next decade this concept evolved into “damage control” which was a major paradigm shift for trauma surgeons [37-39]. This practice became standard of care worldwide by the mid-1990s and has saved the lives of many patients who previously exsanguinated on the OR table. However, the role of *DCL* in emergency general surgery is controversial [40-43]. It is often confused with the concept of a *planned relaparotomy* (described above). Moore et al. proposed that the purpose of *DCL* in intra-abdominal sepsis is different from trauma. While the “bloody viscous cycle” can occur with intra-abdominal sepsis, exsanguination is uncommon short of technical mishaps. Rather patients with intra-abdominal sepsis can present in persistent septic shock [40]. Initially, they are too unstable to undergo immediate operation. An immediate operation in these patients results in a high risk for postoperative acute kidney injury (AKI) sets the stage for MOF, prolonged intensive care unit (ICU) stays and dismal long-term outcomes [40,44,45]. By their protocol, patient presenting in septic shock warrant pre-operative optimization with early goal directed therapy. If they are not optimized pre-operatively, they will experience profound hypotension when subjected to general anesthesia and require high doses vasopressors (typically boluses of phenylephrine) to maintain mean arterial pressure (MAP) and if they undergo a traditional *HP* this will be prolonged and contribute substantially to post-operative AKI [45]. After optimization (described below), the patient is taken to the OR. After undergoing general anesthesia, the surgeon assesses whether the patient is still in septic shock. If so, the OR team is informed that a *DCL* is going to be performed. They should anticipate a short operation (roughly 30–45 minutes) and get the supplies necessary for a *TAC*. A limited colon resection of the inflamed perforated colon is performed using staplers (referred to as a “*perforection*”) with no colostomy and a *TAC* is performed using a “vac pack” technique. The patient is returned to the ICU for ongoing resuscitation. Once physiologic abnormalities are corrected, the patient is returned to the OR for peritoneal lavage and colostomy formation. A definitive resection should be done if feasible for patients who have undergone a limited resection at the previous *DCL* to prevent a fistula and recurrence. However, Kafka-Ritsch et al. propose an alternative reason to perform *DCL* in patients with diverticulitis is to avoid a colostomy by performing a delayed anastomosis [43]. In a prospective study 51 patients with perforated diverticulitis (stage III/IV) were initially managed with limited resection, lavage and *TAC* with a vacuum-assisted closure device followed by second, reconstructive operation 24–48 hours later supervised by a colorectal surgical specialist. Bowel continuity was restored in 38 (84%) patients, of which four were protected by a loop ileostomy. Five anastomotic leaks (13%) were encountered requiring loop ileostomy in two patients or *HP* in three patients. Postoperative abscesses were seen in four patients, abdominal wall dehiscence in one and relaparotomy for drain-related small bowel perforation in one. The overall mortality rate was 10% and 35/46 (76%) of the surviving patients left the hospital with reconstructed colon continuity. Fascial closure was achieved in all patients.

### Summary

Over the last century, based primarily on retrospective case series, we have seen a progression in the treatment of perforated diverticulitis from a conservative 3 stage procedure in the 1940s to the 2 stage *HP* in the 1980s (which is practiced by many surgeons today) and most recently an aggressive one stage *PRA* that is being promoted by colorectal surgical specialists. However, now there is emerging evidence that we should adopt a minimalist strategy of *LLD* or *NOM* in the less sick patients while employing *DCL* in the sickest patients. Unfortunately, like most of the literature on diverticulitis, these recent studies are retrospective and we are awaiting the results of PRTs that are ongoing in Europe [46,47]. Given this lack of high grade data, we propose a reasonable treatment algorithm based on the expert opinion of surgeons who actively practice emergency surgery [40,47-49].

## Decision making algorithm

Key Questions that drive decision making include:

1) Is clinical diagnosis consistent with perforated sigmoid diverticulitis?

2) Does the patient require an emergency operation?

3) Is the patient in septic shock and should undergo pre-operative optimization?

4) Is the patient in septic shock and should undergo damage control laparotomy?

5) Should the patient undergo laparoscopic lavage and drainage?

6) What is a definitive resection and should the patient undergo colostomy or a primary anastomosis?

7) Should the patient undergo interventional radiologic percutaneous drainage?

8) Should the patient be observed and what constitutes observational therapy?

9) Should patients undergo delayed colonoscopy after acute diverticulitis to rule out colon cancer?

10) Should patients with perforated sigmoid diverticulitis who respond to conservative therapy undergo delayed elective colon resection?

11) Should patients after a Hartmann’s Procedure have a colostomy closure and what is the optimal time?

**Figure 2:**
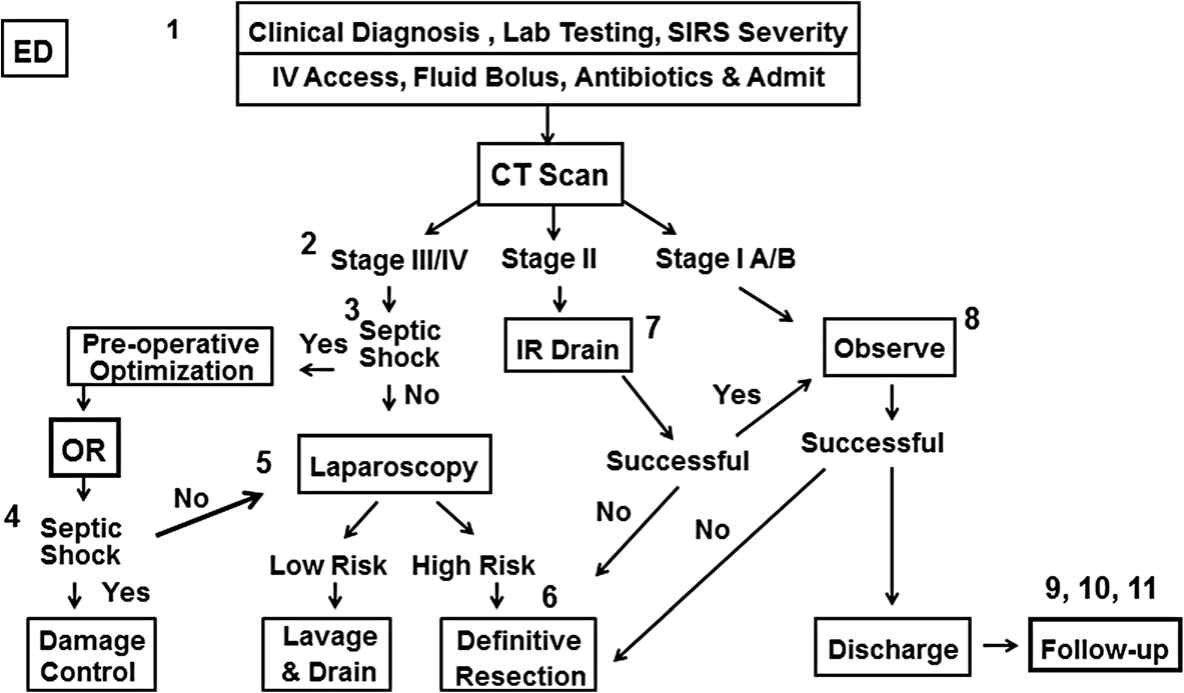
Decision making algorithm for perforated sigmoid diverticulitis.

Figure 2 depicts our proposed management algorithm for acute complicated diverticulitis.

### Making the clinical diagnosis

When encountering a new patient in the emergency department (ED), the surgeon first makes the clinical diagnosis of diverticulitis based on history, physical exam and routine laboratory testing. Abdominal pain is the primary presenting symptom. It is typically located in the left lower quadrant; however, a redundant sigmoid colon can reach the right lower quadrant and mimic appendicitis. Localized peritoneal irritation can result in guarding and rebound tenderness. Free perforation often presents as frank peritonitis. Fever and leukocytosis are usually present and assist in making the clinical diagnosis. Nausea and vomiting are the most notable symptoms when a stricture results in an obstruction. The initial assessment should include a) an assessment of the severity of the signs of the systemic inflammatory response syndrome (SIRS) including heart rate, respiratory rate, temperature and white blood cell count, b) peritonitis on physical exam and c) signs of organ dysfunctions. Patients with clinical diagnosis consistent with diverticulitis who have concerning signs of sepsis should be considered to be at high risk for complicated diverticulitis. They should have IV access obtained, be given a bolus of IV isotonic crystalloids (20 ml/kg), be administered IV antibiotics, and be admitted to the hospital.

These patients should undergo CT scanning with IV contrast of the abdomen and pelvis with the exception of pregnant women where ultrasound is recommended [50]. CT scanning has a high sensitivity and specificity in confirming the diagnosis and identifying patients who are candidates for therapeutic *PCD*[51,52]. CT scanning also excludes other causes of left lower quadrant abdominal pain (e.g. leaking abdominal aortic aneurism or an ovarian abscess), but is not reliable in differentiating acute diverticulitis from colon malignancy [53].

### Patients who require an emergency operation

This decision mostly pertains to patients with stage III and stage IV diverticulitis who present with signs of sepsis and need an emergency operation for source control. The timing and type of source control is unclear. Traditionally, all of these patients were taken expediently to the OR. However, there has been a shift in this paradigm with the recognition that operating in the setting of septic shock sets the stage for postoperative AKI, MOF, prolonged ICU stays and dismal long-term outcomes [40,44,45]. Specifically, we believe patients in septic shock benefit from *pre-operative optimization*. This takes 2–3 hours [54,55]. It starts with obtaining two large bore IV lines through which broad spectrum antibiotics and a bolus of isotonic crystalloids (20 ml/kg) are administered. A central line (via the internal jugular vein placed under ultrasound guidance) and an arterial line are concurrently placed. With ongoing volume loading, CVP is increased to above 10 cmH_2_O. At this point the patient is intubated and ventilation optimized. Norepinephrine is titrated to maintain MAP >65 mm Hg and if high doses are required, stress dose steroids and low dose vasopressin are administered. Electrolyte abnormalities are corrected and blood products are administered based on institutional guidelines. Lactate and mixed venous hemoglobin saturations are measured and trended to assess the adequacy of the resuscitative efforts. Once the patient is stable enough to tolerate OR transport and general anesthesia, he/she should be transported to the OR for a source control operation. After the patient is in the OR and under general anesthesia, the surgeon needs to reassess whether the patient is still in septic shock. If so, the OR team should be informed that a *DCL* is going to be performed (described above). They should anticipate a short operation (roughly 30–45 minutes) and get the supplies necessary for a *TAC*. While the role of *DCL* in this setting is controversial, it should not be confused with the concept of a *planned relaparotomy* (described above) [32]. At the second operation, we believe that the decision to perform a delayed anastomosis should be individualized based on the current physiology, the condition of bowel, patient co-morbidities, and surgeon experience. However, in most patients who have undergone *DCL* because of persistent septic shock, bowel wall edema and persistent hypoperfusion make a delayed anastomosis an unsafe option.

For patients who have stage III and stage IV disease and concerning signs of sepsis but are not in septic shock also need source control. While traditionally these patients were taken expeditiously to the OR for a *HP* or a *PRA*, we believe that the recent case series indicate that *LLD* is a viable option that should be employed to low risk patients but recommend a definitive sigmoid resection for high risk that include patients who are a) immunocompromised, b) have severe co-morbidities c) organ dysfunctions attributable to ongoing sepsis or d) stage IV disease. The again the decision to perform an anastomosis should be individualized based on the current physiology, the condition of bowel, patient co-morbidities, and surgeon experience.

### Patients who do not require an emergency operation

Initial recommended treatment of stage IA and IB diverticulitis includes a) nil per os (NPO), b) nasogastric tube to treat (if present) symptoms of nausea, vomiting and abdominal distention and c) antibiotics with activity against common gram-negative and anaerobic pathogens. A number of single agents and combination regimens provide such activity. However, there is little evidence on which to base selection of specific antimicrobial regimens, and no regimen has demonstrated superiority [56,57]. In general, episodes of diverticulitis severe enough to warrant hospitalization should be initially managed with IV antibiotics. Oral antibiotic therapy can be started when the patient's condition improves and continued as outpatient treatment. There is a paucity of data regarding the optimal duration of antimicrobial therapy.

Patients with stage II diverticulitis should be managed as above but should also be evaluated by interventional radiology for CT guided *PCD*[51]. The preferred approach is trans-abdominal either anterior or lateral, attempting to avoid the inferior epigastric or deep circumflex iliac vessels. Other approaches include transgluteal, transperineal, transvaginal or transanal. Reported failure rates for PCD range from 15% to 30% with a complication rate of 5% (including bleeding, perforation of a hollow viscous or fistula formation) [58-60].

### Observation

Patients with stage IA, IB and II diverticulitis should be treated as described above and observed with serial a) physical exams, b) assessments of SIRS severity and c) laboratory evidence organ dysfunctions. It is expected that their clinical condition will improve over 72 hours. If it does not improve or their condition worsens they should undergo an urgent operation. Patients who resolve their symptoms should be discharged to home on oral antibiotics with follow-up (described below).

### Patients who fail observation

These patients should undergo definitive sigmoid resection. While laparoscopic colon resection compared to open laparotomy colon resection is associated with better outcomes in elective surgery [61,62], there is no evidence that the same is true in urgent/emergent operations. Definitive sigmoid resection requires mobilization of the sigmoid colon with avoidance of injury to the ureters. Ureteral stents should be used selectively in those patients with abscesses or excessive inflammation in the pelvis. For definitive resection the distal margin of resection should be the upper rectum [63] while the proximal margin of resection should go back to non-inflamed descending colon. All diverticuli do not need to be resected. The splenic flexure is generally not mobilized unless needed to form colostomy when indicated. As previously discussed, the major debate is whether to perform a *PRA* or a *HP*. A variety of factors need to be considered including a) disease severity b) condition of bowel at the site of anastomosis, c) patient physiology, d) nutritional status, e) patient co-morbidities, f) hospital/situational factors and g) surgeon experience. Another unresolved debate is should a protecting diverting ileostomy be added if a *PRA* is performed? Unless conditions are optimal, this is the prudent option. The use of perioperative colonic lavage appears to lower complications with *PRA*, but the supporting evidence is limited [64]. Omentoplasty does not offer any benefits [65]. The inferior mesenteric artery should be preserved when feasible to lower the risk of an anastomotic leak [66].

### Discharge and follow-up

Although there is lack of evidence that lifestyle changes will help prevent recurrent diverticulitis, it is likely that measures thought to prevent an initial episode of diverticulitis would also apply to preventing a recurrence. These healthy lifestyles should be recommended upon discharge and include a) physical exercise, b) a high fiber diet, c) reduced red meat, d) minimize alcohol consumption and e) stop smoking [67,68]. Patients should return to the clinic if symptoms recur and have a follow-up clinic appointment at four to six weeks to address three issues.

#### Colonoscopy

After the inflammation from a new onset of diverticulitis has resolved, traditionally patients have undergone colonoscopy to rule out colon cancer. However, the need for routine colonoscopy has recently been questioned [69]. Colonoscopy is a time-consuming and a resource burden on an already-stretched health care system. In addition, endoscopy may be technically more difficult in these patients with an risk iatrogenic bowel perforation (~0.1%). The reported incidence of colon cancer in CT diagnosed acute diverticulitis ranges from 0.5 to 3%. But with technological improvement in quality and resolution of CT has led to better evaluation of the colon in the affected segment and the chances of missing a colon cancer has decreased. A recent study by Sallinen et al. provides additional insight into this debate [70]. They looked 536 patients were admitted to the hospital for diverticulitis who were treated without an operation. Of these patients 394 underwent a delayed colonoscopy and 17 (2.7%) were found to have cancer. Sixteen cancer cases (94%) had abscess in the CT, whereas the remaining case had pericolic extraluminal air, but no abscess. Of the patients with abscess, 11% had cancer mimicking acute diverticulitis. No cancer was found in patients with uncomplicated diverticulitis. Besides abscess, other independent risk factors for cancer included suspicion of cancer by a radiologist, thickness of bowel wall over 15 mm, no diverticula seen, and previously undiagnosed metastases. They conclude that routine colonoscopy after CT-proven uncomplicated diverticulitis seems unnecessary. However, colonoscopy should be performed in patients diagnosed with a diverticular abscess or those with one of the independent risk factors. Barium enema or CT colonography can be used in cases where a complete colonoscopy cannot be accomplished.

#### Prophylactic sigmoid colectomy

In the recent past, a delayed elective sigmoid resection was recommended after two cases of uncomplicated or one case of complicated acute diverticulitis [23]. The idea was that the elective resection would be less morbid than a recurrent bout of diverticulitis. However, an elective resection has risks including a) up to 10% recurrence, b) 1-2% mortality and c) a 10% need for a stoma. Additionally, it is now apparent that the majority of patients with severe diverticulitis present at their 1^st^ episode and that recurrent diverticulitis is relatively rare (roughly 2% per year). Additionally, when it recurs it is less likely to require an operation and has a very low mortality. As a result the indications for elective resection after acute diverticulitis have changed substantially [67,68,71-74]. The following is a recommended list:

a) a Elective resection should be done after one documented episode acute diverticulitis in patients with one or more of the following risk factors including immunosuppression, chronic use of steroids, chronic renal failure, diabetes mellitus, COPD, or collagen vascular disease.

b) For patients without the above risk factors, the preferred timing of elective surgery is after the 3^rd^ or 4^th^ episode of uncomplicated diverticulitis.

c) Patients with one episode of complicated diverticulitis with persistent or recurrent symptoms.

d) Patients with complicated diverticulitis who have an anatomic deformity including a stricture or fistula.

The timing of this elective colectomy is debated but generally one waits 4–6 weeks to allow the inflammation to subside [75,76]. Laparoscopic colectomy is preferred open colectomy [61,62].

### Colostomy closure

For patients who have undergone a *HP*, colostomy closure is performed in only about half of the patients [25,77]. Many of the patients are elderly with multiple risk factors that contraindicate a second surgical procedure. Additionally, colostomy closure carries significant risk of peri-operative complications (10 to 40%) [78]. Patients who are satisfied with living with a colostomy may not want assume these risks as well as the time and the expense of a second operation. The optimal timing colostomy closure it not clear [79,80]. It should not be performed until the patient has resolved their acute phase response and resolved nutritional deficiencies to optimize wound healing reducing the risk of anastomotic leak and wound infection. This usually takes three to six months but sometimes up to a year or never. It depends of the patient’s age, co-morbidities and how deconditioned they were at the time of hospital discharge. Recent studies have documented that the long-term outcomes of elderly patients after being hospitalized for sepsis is notably poor [81,82].

## Conclusion

Based on available clinical data and our collective expert opinions, we propose a management strategy that we feel is rational and safe. All patients with presumed complicated diverticulitis should undergo CT scanning with IV contrast. This will confirm the clinical diagnosis and allow staging of the disease. Therapeutic decision in the based on a) stage of disease, b) patient co-morbidity and c) sepsis severity. Patients with stage I/II disease generally do not present with severe sepsis/septic shock (SS/SS) and can be safely treated with bowel rest, IV antibiotics and *PDC* of larger abscesses. If stage I/II the fail *NOM* or progress into SS/SS they should undergo *PRA* or *HP* depending a variety factors outlined above. Patients with stage III/IV disease may present in septic shock. If so they should undergo pre-operative optimization and if septic shock persists once in the operating room (OR), they should undergo *DCL* with a limited resection. If conditions are optimal at 2^nd^ OR a delayed *PRA* should be performed. If condition are unfavorable, and *HP* should be done. If patients stage III/IV do not present in septic shock they should be taken to the OR and undergo laparoscopy. Low risk patients should undergo *LLD* while high risk patients [i.e. a) immunocompromised, b) have severe co-morbidities c) organ dysfunctions attributable to ongoing sepsis or d) stage IV disease] should undergo *PRA* or *HP* depending a variety factors outlined above. Proximal diverting ileostomy should be used liberally with *PRA*.

## Abbreviations

CT: Computerized tomographic; CVP: Central venous pressure; ED: Emergency room; EBG: Evidence based guideline; DCL: Damage control laparotomy; HP: Hartmann’s procedure; IV: Intravenous; LLD: Laparoscopic lavage and drainage; MAP: Mean arterial pressure; MOF: Multiple organ failure; NOM: Nonoperative management; OR: Operating room; PCD: Percutaneous drainage; PRA: Primary resection anastomosis; PRTs: Prospective randomized trials; SS/SS: Severe sepsis/septic shock; TAC: Temporary abdominal closure.

## Competing interests

The authors have no competing interests and nothing to disclose.

## Authors’ contributions

All of the authors (FM FC,EM, AL, and AP) have a) made substantial contributions to conception and design of this position paper, b) been involved in acquisition of relevant references and their interpretation; c) been involved in drafting the manuscript or revising it critically for important intellectual content; d) given final approval of the version to be published; and e) agree to be accountable for all aspects of the work in ensuring that questions related to the accuracy or integrity of any part of the work are appropriately investigated and resolved. All authors read and approved the final manuscript.
